# Bone density of the femoral neck in patients on maintenance dialysis

**DOI:** 10.1371/journal.pone.0197965

**Published:** 2018-05-24

**Authors:** Kazushige Nakanishi, Kanji Shishido, Chiaki Kumata, Kae Ito, Yutaka Nakashima, Mikio Wakasa

**Affiliations:** 1 Internal Medicine, Sekishin-kai Kawasaki Clinic, Kawasaki-shi, Kanagawa, Japan; 2 Department of Food Science and Nutrition, Showa Women’s University, Setagaya-ku, Tokyo, Japan; The University of Tokyo, JAPAN

## Abstract

**Background:**

Our institution recently started using the femoral neck (FN), as well as the non-shunted distal radius (Rd), to measure bone mineral density (BMD) in patients with chronic kidney disease. We examined the utility and characteristics of this measurement in patients on maintenance dialysis.

**Methods:**

We selected 293 patients on chronic dialysis. We measured Rd and FN BMD using dual-energy X-ray absorptiometry, and we reviewed blood test findings, which included hemoglobin, albumin, blood urea nitrogen, creatinine, adjusted calcium, phosphorus, alkaline phosphatase, and intact parathyroid hormone. We conducted a multiple linear regression analysis that was stratified according to sex, age, body weight, height, and dialysis vintage. The Rd and FN BMD values were the dependent variables, and the blood test findings were the independent variables. We compared the areas under the curve (AUCs) of Rd and FN BMD using receiver operating characteristic curve analysis to differentiate between patients with and without fractures.

**Results:**

FN BMD was significantly lower than Rd BMD. The general risk factors for osteoporosis, such as low body weight, older age, muscle mass loss, and malnutrition, influenced FN BMD. FN and Rd BMD were not correlated with calcium, phosphorous, or intact parathyroid hormone, whereas a significant, negative correlation with alkaline phosphatase was detected. Both men and women with a history of fragility fractures had significantly lower Rd and FN BMDs than patients without such a history. However, there was no significant difference between the AUCs of FN and Rd BMD for fractures in both men and women.

**Conclusions:**

FN BMD was significantly lower than Rd BMD. Additionally, FN BMD was not inferior to Rd BMD for assessing the risk of fracture in patients on maintenance dialysis.

## Introduction

In general, osteoporosis is considered the greatest risk factor for bone fractures, and chronic kidney disease (CKD) is an independent risk factor for osteoporosis, even when traditional risk factors are absent [1]. In addition, it is widely believed that osteoporosis-induced bone fractures, particularly hip fractures, decrease the patient’s quality of life and increase mortality rate, because bone fractures are directly related to life expectancy [2–5]. Bone mineral density (BMD) in patients receiving chronic dialysis is substantially lower than that in healthy subjects of similar age due to CKD-mineral and bone disorder (CKD-MBD). Therefore, the occurrence of bone fractures is substantially higher in patients undergoing chronic dialysis than in the general population [6]. In Japan, the incidence of proximal femoral fractures in patients on maintenance dialysis is about five times that of the general population [7], but no countermeasures have been established albeit the need for aggressive therapeutic intervention. As such, it is clear that reducing the risk of hip fracture could positively contribute to the life expectancy of patients on dialysis. Therefore, the prevention of hip fracture is an important issue that needs to be addressed in dialysis medical care.

BMD measurement is valid for the risk assessment of bone fractures, and there is a strong correlation between BMD and the occurrence of hip fractures among patients without CKD [8–10]. A meta-analysis of patients on dialysis described that there was a significant relationship between low BMD of the lumbar spine and radius and bone fractures [11]. On the other hand, some reports showed that BMD does not predict the risk of fracture in patients receiving hemodialysis [12–14]. However, there is growing evidence that BMD can predict fractures in patients with CKD stage 5D [15,16]. In addition, the updated Kidney Disease: Improving Global Outcomes guideline on CKD-MBD suggests BMD testing to assess the risk of fracture in patients with CKD G3a–G5D with evidence of CKD-MBD and/or risk factors of osteoporosis, if the results will impact treatment decisions [17].

Parathyroid hormone (PTH) causes a strong decline in the BMD of cortical bone compared to that of cancellous bone in patients with hyperparathyroidism. Furthermore, a decline in BMD is also prominently found in patients with cortical bone-based distal radius (Rd) BMD, and measurement of the radius is generally considered appropriate for patients receiving dialysis [18]. At our medical institution, periodic bone densitometry of the Rd has been conducted using dual-energy X-ray absorptiometry (DXA). In addition, measurements from both the lumbar spine and femur are recommended, according to the diagnostic criteria for osteoporosis. However, evaluating the BMD of the lumbar spine in patients receiving dialysis may be difficult because of arteriosclerosis and other factors. Furthermore, a decline in femoral neck (FN) BMD may be related to a significant increase in mortality in the general population [19,20]. In addition, FN BMD is considered useful for predicting proximal femoral fracture because the predictive ability of bone fracture is considered high when the BMD measurement is obtained at the same site as the fracture. Therefore, our medical institution has recently started using the FN, in addition to the Rd, to measure BMD. In this study, we examined the usability and characteristics of these measurements, while comparing Rd and FN BMD via DXA in patients on maintenance dialysis.

## Materials and methods

This study was approved by the Ethics Review Committee at Showa Women’s University, and was conducted in compliance with the ethical principles of the Declaration of Helsinki.

We selected 289 patients who were undergoing chronic dialysis as study subjects (195 men, 94 women; mean age: 63 years; mean time on dialysis: 10 years; 93 patients had diabetes; 271 patients received vitamin D therapy). We measured Rd and FN BMD using DXA (Hologic, Inc., Newark, DE, USA) between March 2014 and April 2015 at the Kawasaki Clinic. Then, we comparatively reviewed blood test findings (hemoglobin [Hb; g/dl], albumin [Alb; g/dl], blood urea nitrogen [mg/dl], creatinine [Cr; mg/dl], adjusted calcium [mg/dl], phosphorus [P; /dl], alkaline phosphatase [ALP; U/L], and intact PTH [intPTH; pg/ml]) from the same month as DXA. In addition, a fragility fracture was defined as: (1) a vertebral or hip fracture and (2) other fractures and a young adult mean (YAM) of 80% or lower in the proximal portion of the forearm bone, humerus, rib, pelvis, and tibia.

We conducted a multiple linear regression analysis that was stratified according to sex, age, body weight, height, presence of diabetes mellitus, and dialysis vintage. Rd and FN BMD values were used as dependent variables, and using the variable reduction method for variable selection, the blood test findings were used as independent variables. Additionally, we compared the areas under the curve (AUCs) for Rd and FN BMD using receiver operating characteristic (ROC) curve analysis to distinguish between patients with and without fractures.

Data were analyzed using SPSS version 25 software (IBM SPSS, New York, NY, USA). For statistical comparisons, we used the Student’s *t*-test, Mann–Whitney U test, McNemar’s test, or chi-squared test according to the characteristics of the data. For each test, the significance level was set at 0.05.

## Results

Subject demographics, blood test values, and BMD are shown in Table 1. BMD is also presented as the comparative YAM percentage (%) because YAM% is used as a guideline for the diagnosis and treatment of osteoporosis in Japan, and 70% of the YAM corresponds to a T-score of -2.5 (standard deviation). The average results were 88.9% for Rd YAM% in men, 74.9% for FN YAM% in men, 75.6% for Rd YAM% in women, and 63.9% for FN YAM% in women. FN YAM% was significantly lower than Rd YAM% in all patients, and in men and women. In addition, men had a significantly greater YAM% for both FN and Rd than women. The percentage of patients with a YAM% less than 70% of the FN was 39.5% for men and 64.9% for women. The percentage of patients with a YAM% less than 70% of the Rd was 7.2% for men and 42.6% for women. And there were significant differences between them (Table 1).

**Table 1:** Demographics, biochemical parameters, and BMD.

	All (n = 289)	Men (n = 195)	Women (n = 94)	P value
Age(yrs)	63.07±13.01	61.18±12.85**	67.0±12.51	<0.01
Dialysis vintage (y)	10.43±9.76	9.59±9.07	12.17±10.90	0.098
Body weight(kg)	58.65±13.92	63.13±13.38**	49.349±9.86	<0.01
Height(cm)	160.63±9.92	165.56±6.91**	150.40±6.95	<0.01
Diabetes (%)	32.2	33.8	28.7	0.382
Hb (g/dl)	10.73±0.88	10.73±0.91	10.72±0.84	0.637
Alb (g/dl)	3.69±0.35	3.72±0.37*	3.64±0.30	0.037
BUN (mg/dl)	63.55±15.10	64.07±15.73	62.44±13.68	0.599
Cr (mg/dl)	11.18±2.90	11.98±2.79**	9.54±2.41	<0.01
adjCa (mg/dl)	8.92±0.55	8.90±0.53	8.94±0.54	0.564
P (mg/dl)	5.47±1.20	5.49±1.24	5.45±1.14	0.637
ALP (U/L)	258.73±154.3	232.44±84.99**	313.28±232.81	<0.01
Intact PTH (pg/ml)	212.62±143.96	213.99±143.68	209.78±145.32	0.633
FN YAM%	71.46±16.09†	75.24±15.39**†	63.66±14.73†	<0.01
Rd YAM%	85.21±16.87	89.66±13.98**	75.98±18.60	<0.01
FNYAM%<70 (%)	47.8‡	39.5**‡	64.9‡	<0.01
RdYAM%<70 (%)	11.3	7.2**	42.6	<0.01
Fragility fracture (%)	14.2	10.8*	21.3	0.016

Data are presented as mean±standard deviation or percentage. Hb, hemoglobin; Alb, albumin; BUN, blood urea nitration; Cr, creatinine; adjCa, adjusted calcium; P, phosphorous; ALP, alkaline phosphatase; PTH, parathyroid hormone; YAM, young adult mean; FN, femoral neck; Rd, non-shunted distal radius.

*P<0.05 vs female group;

**P<0.01 vs female group;

†P<0.01 vs RdYAM%;

‡P<0.01 vs RdYAM%<70

**Fig 1:**
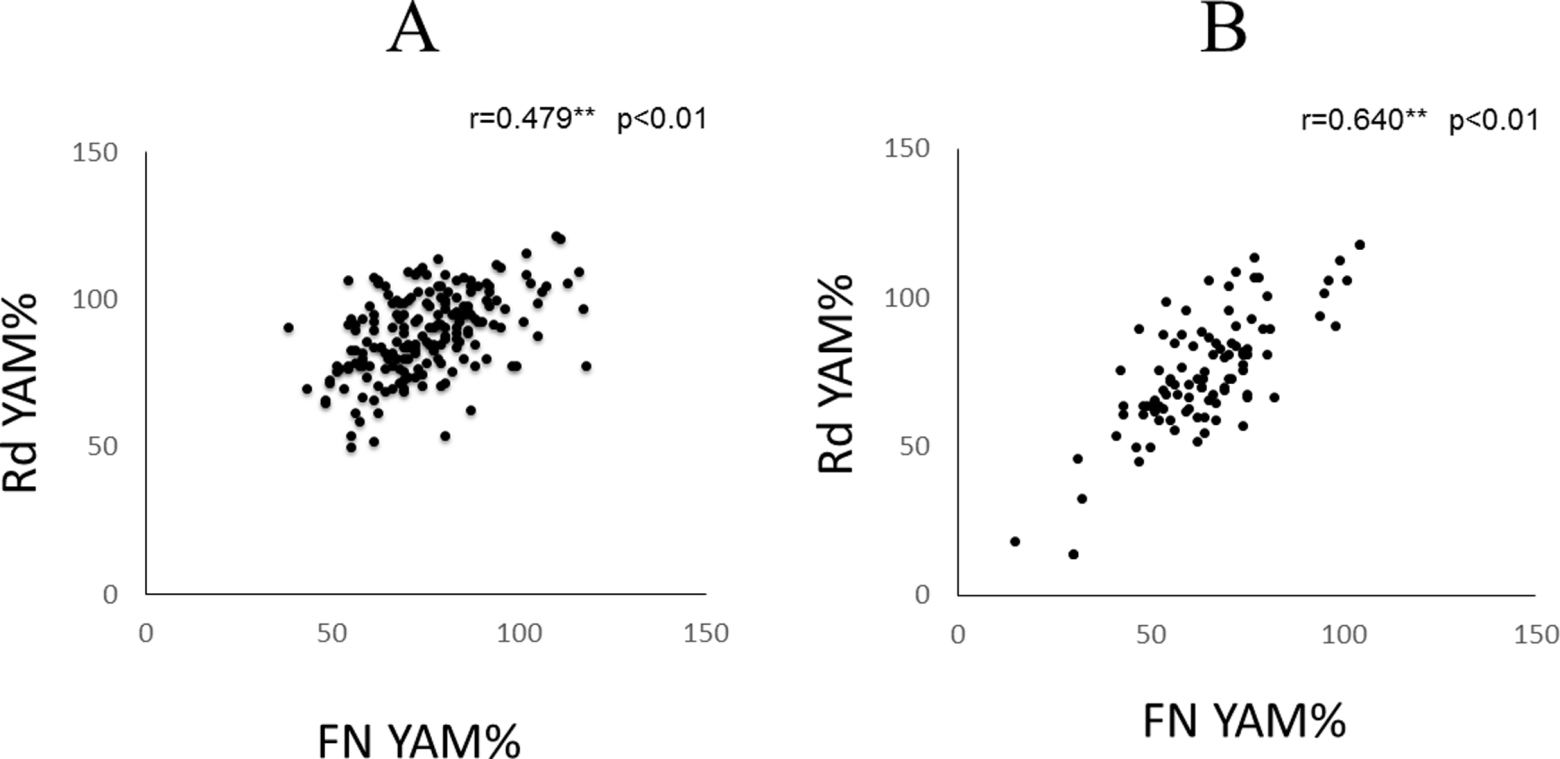
**Scatterplot of BMD in (A) men and (B) women on maintenance dialysis.** YAM, young adult mean; Rd, non-shunted distal radius; FN, femoral neck.

There were significant positive Spearman’s rank correlations between FN YAM% and Rd YAM% for both men and women (Fig 1).

Correlations between BMD and age, dialysis vintage, height, and body weight were respectively evaluated using Spearman’s rank correlations (Table 2). Both FN and Rd BMD had significant correlations with age, height, and body weight. For dialysis vintage, a negative correlation was detected with the Rd, but not with the FN. The correlations between blood test values and BMD are also shown in Table 2. There were positive correlations between BMD and Hb, Alb, and Cr, but there was no correlation with P and intPTH. Conversely, there was a significant negative correlation with ALP for both FN and Rd BMD.

**Table 2:** Correlations between YAM% and characteristics and biochemical parameters.

	BMD			
	Mean FN YAM%		Mean Rd YAM%	
	r	P value	r	P value
Age (yrs)	-0.41	<0.01	-0.431	<0.01
Dialysis vintage (y)	-0.037	0.526	-0.306	<0.01
Height (cm)	0.465	<0.01	0.479	<0.01
Body weight (kg)	0.542	<0.01	0.497	<0.01
Hb (g/dl)	0.178	<0.01	0.174	<0.01
Alb (g/dl)	0.208	<0.01	0.163	<0.01
Cr (mg/dl)	0.41	<0.01	0.398	<0.01
adjCa (mg/dl)	-0.117	<0.05	-0.126	<0.05
P (mg/dl)	0.143	<0.05	0.097	<0.1
Intact PTH (pg/ml)	-0.007	0.909	-0.006	0.913
ALP (U/L)	-0.283	<0.01	-0.303	<0.01

BMD, bone mineral density; YAM, young adult mean; FN, femoral neck; Rd, non-shunted distal radius; r, Spearman’s rank correlation coefficient; Hb, hemoglobin, Alb, albumin; Cr, creatinine; adjCa, adjusted calcium; P, phosphorous; PTH, parathyroid hormone; ALP, alkaline phosphatase.

The results of the multiple linear regression analysis are shown in Table 3. Age was the strongest negative independent variable associated with Rd BMD (β = -0.487, P < 0.001) in women, followed by dialysis vintage (β = -0.297, P < 0.001), serum Cr (β = 0.241, P = 0.011), and ALP (β = -0.158, P = 0.039). Similarly, age was the strongest negative independent variable associated with FN BMD (β = -0.422, P < 0.001) in women; body weight (β = 0.291, P < 0.001), serum Cr (β = 0.205, P = 0.020), and Alb (β = 0.167, P = 0.021) were also significantly associated with Rd BMD in women. In men, body weight was the strongest independent variable associated with FN BMD (β = 505, P < 0.001), followed by adjusted calcium (β = -0.147, P = 0.017) and diabetes mellitus (β = -0.131, P = 0.032). However, age was not significantly associated with FN BMD in men. Dialysis vintage was the strongest negative independent variable associated with Rd BMD (β = -0.319, P < 0.001) in men, with age (β = -0264, P < 0.001), body weight (β = 0.235, P < 0.001), intPTH (β = -0.167, P = 0.008), and adjusted calcium (β = -0.121, P = 0.047) also significantly associated.

**Table 3:** Multipls regression analysis.

		Regressor (Constant)	B	Β	P values	95% confidence interval		Adjusted R (ANOVA-p)
						Lower	Upper	
FN	Men	(77.65)						0.283 <0.001
		body weight	0.581	0.505	<0.001	0.443	0.72	
		adjCa	4.231	-0.15	0.017	-7.7	-7.6	
		DM	-4.25	-0.13	0.037	-8.149	-0.354	
	Women	(33.912)						0.566 <0.001
		age	0.497	-0.42	<0.001	-0.697	-0.298	
		body weight	0.435	0.292	<0.001	0.216	0.655	
		Cr	1.253	0.205	0.02	0.2	2.3	
		Alb	8.12	0.167	0.021	1.28	14.97	
Rd	Men	(94.188)						0.276 <0.001
		dialysis vintage	0.507	-0.33	<0.001	-0.698	-0.317	
		age	0.262	-0.24	<0.001	-0.405	-0.12	
		body weigh	0.248	0.239	0.01	0.109	0.387	
	Women	(116.284)						0.481 <0.001
		age	0.722	-0.49	<0.001	-0.993	-0.451	
		dialysis vintage	0.505	-0.3	<0.001	-0.761	-0.249	
		Cr	1.865	0.243	0.011	0.437	3.293	
		ALP	0.013	-0.16	0.039	-0.025	-0.001	

B, partial regression coefficient; β, standardized partial regression coefficient; R^2^, coefficient of determination; FN, femoral neck; adjCa, adjusted calcium; DM, diabetes mellitus; Cr, creatinine; Alb, albumin; Rd, non-shunted distal radius; ALP, alkaline phosphatase.

**Fig 2:**
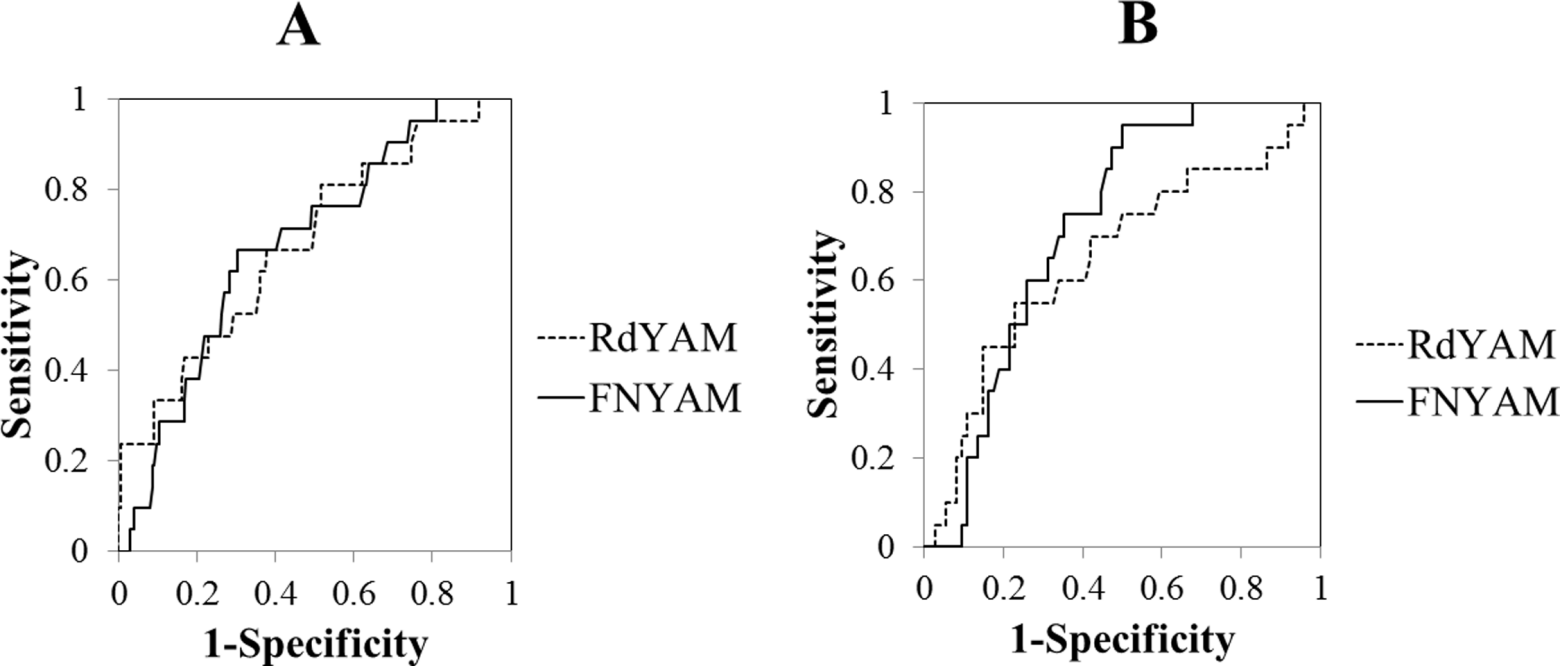
**ROC curves in (A) men and (B) women on maintenance dialysis.** YAM, young adult mean; Rd, non-shunted distal radius; FN, femoral neck.

When the medical history of fragility fractures was considered, we identified 21 (10.8%) men and 20 (21.3%) women who had previously experienced fragility fractures (Table 1), and both men and women with a history of fragility fractures had significantly lower Rd and FN BMDs than patients without such a history (Table 4). To assess the sensitivity-specificity profiles of BMD for fractures, we used ROC curves (Fig 2). All tested parameters showed AUCs that significantly differed from an AUC = 0.5, which represents the line of equality. However, the AUC of the FN was not significantly different from that of the Rd in both men and women (Table 5 and Fig 2).

**Table 4:** BMD of fragility fractures in men and women on maintenance dialysis.

	Men (n = 195)		Women (n = 94)	
Fragility fracture	(-) (n = 174)	(+) (n = 21)	(-) (n = 74)	(+) (n = 20)
RdYAM%	91±13	80±17**	77±19	70±17*
FNYAM%	76±16	67±11**	66±16	56±7**

Data are presented as mean±standard deviation. YAM, young adult mean; Rd, non-shunted distal radius; FN, femoral neck.

*significantly different at P < 0.05;

**significantly different at P < 0.01.

**Table 5:** ROC AUCs for fractures in men and women on maintenance dialysis.

	Parameter	AUC	SE	P*	95%CI	p**
Men	RdYAM%	0.681	0.064	0.005	0.555–0.807	0.268
	FNYAM%	0.681	0.058	0.002	0.567–0.795	
Women	RdYAM%	0.650	0.074	0.044	0.504–0.796	0.998
	FNYAM%	0.723	0.054	<0.001	0.618–0.828	

ROC, receiver operating characteristic; AUC, area under the curve; SE, standard error of the mean; 95%CI, 95% confidence interval; Rd, non-shunted distal radius; YAM, young adult mean; FN, femoral neck.

*comparison between calculated AUC and the line of equality (AUC = 0.5);

**comparison between Rd and FN AUCs.

## Discussion

In the present study, FN BMD was significantly lower than Rd BMD. The percentage of subjects with a FN YAM less than 70% was 39.5% for men and 64.9% for women (Table 1), which was in accordance with the diagnostic criteria of osteoporosis. For FN BMD, older age, low body weight, muscle mass reduction, and malnutrition were significant factors in women, and low body weight was a significant factor in men (Table 3). On the other hand, dialysis vintage, which was specific to patients on hemodialysis, influenced Rd BMD both in men and women, but such an association was not recognized in FN BMD (Table 3). This finding was consistent with previous reports stating that dialysis vintage influences Rd BMD [21,22]. This phenomenon is often observed in cortical bone, and the ratio of cortical bone is higher in the Rd than in the FN.

Both men and women with a history of fragility fractures had significantly lower Rd and FN BMD than patients without such a history (Table 4). Furthermore, the relative changes in BMD of patients with fractures versus those without fractures were similar between FN and Rd BMD in both men and women, although FN BMD was lower than Rd BMD (Table 4). In addition, no significant difference was found between the AUCs of the FN and Rd in men and women (Table 5). Therefore, it is reasonable to conclude that FN BMD is not inferior to Rd BMD for assessing the risk of fractures, and further studies are needed to determine which site is more effective for measuring BMD in patients on maintenance dialysis.

A high serum ALP value could have a significant correlation with hip fractures in patients receiving dialysis, according to a recent Japanese report [23]. In the present study, a significant negative correlation was detected between ALP and FN and Rd BMD, respectively. Bone resorption largely exceeded bone formation, and bone mass decreased due to high-turnover bone conditions in many of our subjects. Since a poor prognosis may be expected in patients with high serum ALP, it is necessary to provide careful treatment to reduce the risk of fragility fractures.

## Conclusion

Although FN BMD was significantly lower than Rd BMD, FN BMD was not inferior to Rd BMD for assessing the risk of fracture in patients on maintenance dialysis.
